# Separating generalized anxiety disorder from major depression using clinical, hormonal, and structural MRI data: A multimodal machine learning study

**DOI:** 10.1002/brb3.633

**Published:** 2017-02-12

**Authors:** Kevin Hilbert, Ulrike Lueken, Markus Muehlhan, Katja Beesdo‐Baum

**Affiliations:** ^1^ Institute of Clinical Psychology and PsychotherapyTechnische Universität DresdenDresdenGermany; ^2^ Behavioral EpidemiologyTechnische Universität DresdenDresdenGermany; ^3^ Department of PsychologyNeuroimaging CenterTechnische Universität DresdenDresdenGermany; ^4^ Department of Psychiatry, Psychosomatics, and PsychotherapyUniversity Hospital WuerzburgWuerzburgGermany

**Keywords:** classification, generalized anxiety disorder, machine learning, major depression, structural MRI, support vector machine

## Abstract

**Background:**

Generalized anxiety disorder (GAD) is difficult to recognize and hard to separate from major depression (MD) in clinical settings. Biomarkers might support diagnostic decisions. This study used machine learning on multimodal biobehavioral data from a sample of GAD, MD and healthy subjects to differentiate subjects with a disorder from healthy subjects (case‐classification) and to differentiate GAD from MD (disorder‐classification).

**Methods:**

Subjects with GAD (*n* = 19), MD without GAD (*n* = 14), and healthy comparison subjects (*n* = 24) were included. The sample was matched regarding age, sex, handedness and education and free of psychopharmacological medication. Binary support vector machines were used within a nested leave‐one‐out cross‐validation framework. Clinical questionnaires, cortisol release, gray matter (GM), and white matter (WM) volumes were used as input data separately and in combination.

**Results:**

Questionnaire data were well‐suited for case‐classification but not disorder‐classification (accuracies: 96.40%, *p* < .001; 56.58%, *p* > .22). The opposite pattern was found for imaging data (case‐classification GM/WM: 58.71%, *p* = .09/43.18%, *p* > .66; disorder‐classification GM/WM: 68.05%, *p* = .034/58.27%, *p* > .15) and for cortisol data (38.02%, *p* = .84; 74.60%, *p* = .009). All data combined achieved 90.10% accuracy (*p* < .001) for case‐classification and 67.46% accuracy (*p* = .0268) for disorder‐classification.

**Conclusions:**

In line with previous evidence, classification of GAD was difficult using clinical questionnaire data alone. Particularly cortisol and GM volume data were able to provide incremental value for the classification of GAD. Findings suggest that neurobiological biomarkers are a useful target for further research to delineate their potential contribution to diagnostic processes.

## 1 Introduction

Generalized anxiety disorder (GAD) is among the most prevalent anxiety disorders in the general population (Beesdo, Pine, Lieb, & Wittchen, 2010; Kessler, Petukhova, Sampson, Zaslavsky, & Wittchen, 2012) and associated with considerable burden for the individual and the society (Andlin‐Sobocki & Wittchen, 2005; Hoffman, Dukes, & Wittchen, 2008). Concurrent comorbidity with major depression (MD) is high (Kessler, Chiu, Demler, Merikangas, & Walters, 2005) and typically between 40% and 60% (Carter, Wittchen, Pfister, & Kessler, 2001; Hunt, Issakidis, & Andrews, 2002). Previous research has shown both insufficient sensitivity in detecting a GAD patient as a case in real‐world clinical settings and low specificity when separating a GAD diagnosis from MD (Calleo et al., 2009; Wittchen et al., 2002). Wittchen et al. (2002) found that only about two‐thirds of all primary care patients with GAD but no depression were identified by their primary care physician as cases with any mental disorder, whereas case recognition was 85% in patients with comorbid GAD and MD. Only 34% of pure GAD cases and 43% of comorbid GAD cases were diagnosed with GAD. Calleo et al. (2009) reported that only 28% of all elderly GAD patients presenting in specialty medical clinics received a diagnosis of any anxiety or mood disorder and only 1.5% were correctly diagnosed with GAD. In other primary care studies, between 30% and 55% of GAD patients were recognized and correctly diagnosed (Munk‐Jorgensen et al., 2006; Vermani, Marcus, & Katzman, 2011). GAD is particularly difficult to separate from MD: Calleo et al. (2009) report that the number of GAD patients receiving a diagnosis of a depressive disorder is more than twice the number of GAD patients receiving a diagnosis of an anxiety disorder. In primary care settings, GAD recognition is facilitated by further clinical information such as the presence of a higher number of disorder symptoms, the presence of comorbid mental disorders and by patients primarily reporting nonsomatic symptoms to their physician (Wittchen et al., 2002). Additionally, detection of GAD in primary care might be supported using screening measures (Herr, Williams, Benjamin, & McDuffie, 2014) such as the GAD‐7 (Spitzer, Kroenke, Williams, & Lowe, 2006) or the Anxiety Screening Questionnaire (ASQ; Wittchen & Boyer, 1998; Wittchen & Perkonigg, 1997). The correct diagnosis is of vital importance as it largely determines the choice of psychotherapeutic or pharmacologic treatment. Improving the differentiation of GAD and MD during the diagnostic process is therefore essential to support clinical decisions.

The use of biomarkers based on the neurobiological differences in disorders has been proposed as one option for increasing diagnostic accuracy (for a review see Wolfers, Buitelaar, Beckmann, Franke, & Marquand, 2015). A useful biomarker has to provide sufficient sensitivity and specificity to predict a given patient's status on the individual level (Lueken et al., 2016; Savitz, Rauch, & Drevets, 2013). Machine learning algorithms have shown predictive potential for single‐subject diagnostic purposes and may thus support personalized medicine approaches. Supervised machine learning algorithms such as support vector machines (SVM) have been used to investigate the potential use of these biomarkers for separating different disorders based on their neural correlates (Grotegerd et al., 2013; Lim et al., 2013; Lueken, Hilbert, Wittchen, Reif, & Hahn, 2015; MacMaster, Carrey, Langevin, Jaworska, & Crawford, 2014; Ota et al., 2013; Pantazatos, Talati, Schneier, & Hirsch, 2014; Schnack et al., 2014; Serpa et al., 2014; Takizawa et al., 2014). Given that GAD and MD do not only show common but also separate neural correlates (Beesdo et al., 2009; Canu et al., 2015; Etkin & Schatzberg, 2011; Oathes, Patenaude, Schatzberg, & Etkin, 2015), machine learning might also be successfully applied to the problem of recognizing GAD patients and separating them from MD patients. Biomarkers do not have to be restricted, however, to neural information (Boksa, 2013; Singh & Rose, 2009). GAD and MDD have also been associated with altered levels of the stress hormone cortisol (Bhagwagar, Hafizi, & Cowen, 2005; Hek et al., 2013; Hinkelmann et al., 2012; Mantella et al., 2008). Results, however, are heterogeneous probably due to different sampling methods and ‐times or comorbidities.

This study aims to use machine learning on multimodal biobehavioral data from a sample of subjects exhibiting GAD, MD, both disorders, or no disorder. In a first step, supervised machine learning based on SVM was used on the entire sample aiming to detect cases versus noncases, that is subjects with a disorder versus healthy comparison subjects (case‐classification). In the second step, SVM was used on patients only in order to detect GAD, that is differentiate subjects with GAD only or GAD with comorbid MD from those with MD only (disorder‐classification). Clinical questionnaire data, cortisol release, and structural brain data including gray matter (GM) and white matter (WM) volumes were used separately and in combination. Classification based on clinical data was hypothesized to perform well for case‐classification but not for disorder‐classification. Given inconsistent results related to cortisol release in GAD and MD, no specific hypotheses were formulated for the hormonal data. Previous work in GAD and MD alone, however, suggested abnormalities related to cortisol for each of these disorders (for reviews see Dedovic & Ngiam, 2015; Hilbert, Lueken, & Beesdo‐Baum, 2014; Staufenbiel, Penninx, Spijker, Elzinga, & van Rossum, 2013), therefore classification based on hormonal data was expected to perform above chance level for both classification problems. Brain imaging data were hypothesized to perform well for both classification problems and thus provide incremental value for the detection and classification of GAD. GM volume input data were restricted to anatomically defined brain regions repeatedly reported in the GAD and MD literature (reviews and meta‐analyses from Bora, Fornito, Pantelis, & Yucel, 2012; Bora, Harrison, Davey, Yucel, & Pantelis, 2012; Du et al., 2012; Hilbert et al., 2014; Kempton et al., 2011; Lai, 2013; Sacher et al., 2012) as recommended in Chu et al. (2012). As no brain regions were identified that were consistently reported in the GAD and MD literature for WM volume, WM input data were only restricted to anatomically defined WM areas in the brain in general.

## 2 Method

### 2.1 Subjects

A convenience sample of subjects with GAD and/or MD as well as healthy comparison subjects were recruited from the outpatient centre for psychotherapy at the Institute of Clinical Psychology and Psychotherapy at TU Dresden and the general public. Inclusion criteria were a current diagnosis of GAD and/or MD according to DSM‐IV‐TR criteria (APA, 2000) for the clinical groups or no lifetime diagnosis of a mental disorder for the healthy comparison group. Subjects were excluded due to psychotropic medication, a nonremitted diagnosis of substance dependence or smoking of more than 10 cigarettes per day or inability to safely obtain a MRI scan. As a result, *n* = 19 subjects with a diagnosis of GAD (*n* = 12 with comorbid MD), *n* = 14 subjects with a diagnosis of MD without GAD and *n* = 24 healthy comparison subjects were included. Current and lifetime diagnoses were determined using the Munich Composite International Diagnostic Interview (DIA‐X/M‐CIDI; Wittchen & Pfister, 1997) and confirmed by experienced clinicians. Appendix S1 provides an overview about comorbid disorders within the clinical groups. The Penn State Worry Questionnaire (PSWQ; Meyer, Miller, Metzger, & Borkovec, 1990), Beck Depression Inventory‐II (BDI; Beck, Steer, & Brown, 1996), Intolerance of Uncertainty Scale‐12 (IUS‐12; Carleton, Norton, & Asmundson, 2007) and the trait version of the State‐Trait‐Anxiety‐Index (STAI‐T; Spielberger, Gorssuch, Lushene, Vagg, & Jacobs, 1983) were used as additional dimensional measures for characterizing all groups. MRI data of the GAD and healthy subjects included in this analysis have been used previously to investigate structural alterations in GAD (Hilbert et al., 2015). While the previous analysis aimed at informing neurostructural disease models, the present analysis chose a complementary view by testing the predictive value of brain morphology as a putative differential diagnostic marker for the individual patient. The study protocol complied with the ethical standards of the relevant national and institutional committees on human experimentation and with the Declaration of Helsinki as revised in 2008. It was approved by the local ethics committee (EK13012009). Subjects provided written informed consent before participating in the study.

### 2.2 Analysis of demographic and clinical data

Chi‐square tests and univariate analyses of variance were used for the analysis of demographic and clinical data as appropriate. Subsequent post hoc tests were used for pairwise comparisons. The level of significance was set at *p* < .05. SPSS 23 (IBM, New York, NY, USA) was used for all calculations. Clinical questionnaire sum scores of the PSWQ, BDI, IUS‐12, and STAI‐T were subsequently used as input data for classification.

### 2.3 Acquisition and analysis of cortisol data

To determine the cortisol release saliva samples were acquired using Salivettes “code blue” (Saarstedt, Nümbrecht, Germany) at six time points over the course of the experimental procedure, including samples ca. 10 min before scanning, directly before scanning, and after four different MRI scans including three different tasks and a structural scan, covering a total of 100 min. Samples were stored at −20°C until being assayed using a commercial chemiluminescence immunoassay (IBL RE 62011) at the Chair of Biopsychology of the TU Dresden (Prof. Dr. Clemens Kirschbaum). Cortisol values were log‐transformed to reach normal distribution. For an estimation of the total cortisol release we calculated the area under the curve with respect to the ground (Fekedulegn et al., 2007; Pruessner, Kirschbaum, Meinlschmid, & Hellhammer, 2003). One subject was excluded from further analyses due to an incomplete cortisol profile.

### 2.4 Structural MRI data acquisition and preprocessing

Imaging data were acquired on a 3‐Tesla Trio‐Tim MRI whole‐body scanner (Siemens, Erlangen, Germany) with a 12 channel head coil located at the Neuroimaging Center of the TU Dresden. A magnetization‐prepared rapid gradient echo imaging sequence (MPRAGE; TE = 2.26 ms, TR = 1,900 ms, flip angle = 9°, FOV = 256 × 256 mm, matrix = 256 × 256) with 176 slices and an isotonic voxel size of 1 × 1 × 1 mm was used. The VBM8 toolbox (http://www.neuro.uni-jena.de/vbm/download/) for SPM8 (http://www.fil.ion.ucl.ac.uk/spm/software/spm8/) was used to segment all images into GM, WM, and cerebrospinal fluid. Subsequently, the segmented data were modulated and DARTEL‐normalized (Ashburner, 2007) to MNI space. During this step, voxel sizes were changed to 1.5 × 1.5 × 1.5 mm resolution. An 8‐mm full‐width half‐maximum Gaussian kernel was used for smoothing of the data. The resulting images were checked for artefacts and included in the subsequent analyses.

### 2.5 Pattern recognition

A total of eight separate classification analyses were conducted, depending on four input data modalities (clinical scores, cortisol data, GM data, WM data) and two classification problems: First, a classifier was trained to correctly classify subjects from both clinical groups (GAD and MD groups) as cases and subjects from the healthy comparison group as noncases. This included 33 subjects with a disorder and 24 HC subjects. Second, only GAD and MD subjects were used and the classifier was trained to correctly classify subjects according to their diagnostic category as GAD subjects (independently of whether comorbidity was present) or MD subjects. This included 19 subjects with GAD and 14 MD‐only subjects. The following procedure was applied for all separate analyses: clinical questionnaire scores, cortisol release, GM maps, and WM maps were used as input for the PRoNTo toolbox (http://www.mlnl.cs.ucl.ac.uk/pronto/; Schrouff, Rosa, et al., 2013). For the MRI data analyses, an overall mask was used to restrict analyses to voxels for which every subject was able to provide data. An additional region‐of‐interest (ROI) mask restricting analysis to the anterior cingulate cortex (ACC), amygdala, prefrontal and orbitofrontal areas, the putamen and nucleus caudate and the hippocampus and thalamus was applied for classification based on GM data given the recommendation to use feature selection based on prior knowledge if prior knowledge is available (Chu et al., 2012). Please see Appendix S2 for an additional whole‐brain approach. These regions were anatomically defined according to the automated anatomical labeling atlas (aal; Tzourio‐Mazoyer et al., 2002) as implemented in the wfu pickatlas toolbox (Maldjian, Laurienti, & Burdette, 2004; Maldjian, Laurienti, Kraft, & Burdette, 2003). An additional ROI mask restricting analysis to WM according to the talairach daemon as implemented in the wfu pickatlas toolbox (Lancaster, Summerln, Rainey, Freitas, & Fox, 1997; Lancaster et al., 2000; Maldjian et al., 2003, 2004) was applied for classification based on WM data. Given the relative lack of prior studies reporting WM volume data in GAD no additional ROIs were used for WM data. Input data were mean centered and normalized for all analyses. SVMs were used for classification within a leave‐one‐out cross‐validation (LOOCV) framework. Sensitivity, specificity, and balanced accuracy of the resulting classification solution were calculated and permutation tests based on 5,000 iterations were used to assess the level of statistical significance set at *p* < .05. Weight‐maps and rank‐orders of the regional weight averages were calculated for the GM data as described in Schrouff, Cremers, et al. (2013).

Beyond classification based on a single input data modality all available data was also integrated into a single decision on group membership and tested. Weight‐adjusted voting for ensembles of classifiers (WAVE; Kim, Kim, Moon, & Ahn, 2011) weights the results from the single classifiers according to which classifiers performed better on difficult cases (i.e. cases which are often misclassified) and allows for the calculation of classifier‐weights and case‐weights. The classifier‐weights can be used to achieve a final decision. For applying WAVE, however, data in every modality for every subject are needed. The subject with incomplete cortisol data was therefore excluded from all following analyses. As WAVE requires assessing the performance of the classifiers before the resulting weights can be used on a new case, a nested LOOCV framework was applied for the integration of classifiers, thus guaranteeing independence of predictions. Classifiers were trained and tested using LOOCV to assess the performance of each classifier for each subject and derive the classifier‐ and subject‐weights in an inner fold. Afterwards, classifiers and their corresponding weights were used to classify a new subject neither part of the training nor test sets in an outer fold. This procedure was again rotated in a LOOCV scheme. For significance testing of the combined classification, permutation testing was used as well: the classifier‐weights resulting from the inner fold were used on permuted labels and the frequency of resulting predictions that were more accurate than the true prediction were counted. This procedure was done for 5,000 iterations. The *p*‐value was subsequently calculated by dividing the number of better predictions during permutation testing by the number of permutations.

## 3 Results

### 3.1 Sample characteristics

Table 1 depicts the sample characteristics per group. GAD (with and without MD), MD and healthy comparison subjects were comparable regarding sex, age, handedness, and education. They were also comparable in smoking status and overall cortisol release. GAD and MD groups showed significantly higher scores compared to the healthy comparison group in all clinical questionnaires. Clinical groups revealed comparable scores in each questionnaire except the intolerance of uncertainty scale‐12 (Carleton et al., 2007), for which GAD subjects scored significantly higher than MD subjects.

**Table 1:** Sample characteristics. Means (*SD*) except where noted

	HC (*n* = 24)	GAD (*n* = 19)	MD (*n* = 14)	χ^2^/*F* (*df*)	*p*
Sociodemographic characteristics					
Female sex (*n*, %)	17 (70.8)	16 (84.2)	12 (85.7)	1.653 (2)	.438
Right‐handed (*n*, %)	21 (87.5)	17 (89.5)	11 (78.6)	0.875 (2)	.646
Secondary school (*n*, %)	18 (75.0)	12 (63.2)	12 (85.7)	3.551 (2)	.470
Nonsmoker (*n*, %)	22 (95.7)	19 (100.0)	13 (92.9)	1.262 (2)	.532
Age in years (mean, *SD*)	32.25 (9.33)	33.47 (8.90)	29.86 (11.71)	0.553 (2)	.578
Clinical characteristics (mean, *SD*)					
PSWQ^a^	36.00 (9.98)	62.00 (6.22)	57.43 (12.06)	45.461 (2)	<.001
BDI^a^	4.50 (4.82)	21.16 (7.37)	21.36 (9.14)	40.485 (2)	<.001
I US‐12^b^	25.75 (6.72)	41.00 (7.03)	32.71 (9.25)	21.899 (2)	<.001
STAI‐T^a^	33.58 (6.77)	56.16 (7.11)	55.00 (7.04)	70.254 (2)	<.001
Cortisol release					
log‐AUC	199.55 (66.05)	159.85 (38.76)	196.61 (70.30)	2.549 (2)	.088

HC, healthy control group; GAD, generalized anxiety disorder group; MD, major depressive disorder group; PSWQ, Penn State Worry Questionnaire; BDI, Beck Depression Inventory‐II; IUS‐12, Intolerance of Uncertainty Scale‐12; STAI‐T, State‐Trait‐Anxiety‐Index, Trait version; log‐AUC, log‐transformed area under the curve.

a GAD + MDD > controls: *p* < .001.

b GAD + MDD > controls: *p* < .01, GAD > MD: *p* < .01.

### 3.2 Case‐classification

**Figure 1:**
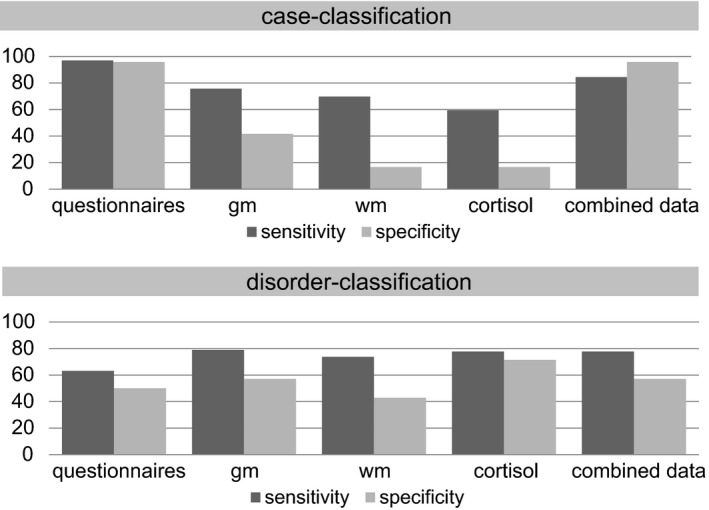
Sensitivity and specificity of case‐classification and disorder‐classification by data modality in percent. Upper half: case‐classification; lower half: disorder‐classification. gm: Gray matter; wm: white matter

Case‐classification using clinical information resulted in almost perfect balanced accuracy (96.40%, *p* < .001, sensitivity: 96.97%, specificity: 95.83%; see Figure 1). Table 2 shows the weighting of the clinical data for classification. This indicates that particularly the STAI‐T scores were important for differentiating cases from noncases. Applying the cortisol data to the same classification problem resulted only in poor balanced accuracy (38.02%, *p* = .84, sensitivity: 59.38%, specificity: 16.67%).

**Table 2:** Weight averages by clinical scores

Rank	Case‐classification			Disorder‐classification		
	Score	Weight abs.	Weight perc.	Score	Weight abs.	Weight perc.
1	STAI‐T	0.7977	45.10	IUS‐12	0.9331	60.58
2	PSWQ	0.4366	24.69	BDI	0.2629	17.07
3	BDI	0.3902	22.06	PSWQ	0.1934	12.55
4	IUS‐12	0.1442	8.15	STAI‐T	0.1508	9.79

GAD, generalized anxiety disorder group; MD, major depressive disorder group; PSWQ, Penn State Worry Questionnaire; BDI, Beck Depression Inventory‐II; IUS‐12, Intolerance of Uncertainty Scale‐12; STAI‐T, State‐Trait‐Anxiety‐Index, trait version; Weight abs., mean of absolute weight values; Weight perc., percentage of weight values of this score relative to the sum of all weight values of all scores for the respective classification problem.

Case‐classification using GM data resulted in a balanced accuracy of 58.71% (*p* = .09, sensitivity: 75.76%, specificity: 41.67%). Table 3 shows the averaged weights of the brain regions according to aal. Particularly the putamen and amygdala were important for case‐classification while areas such as the hippocampus or thalamus were ranked as comparably less important for both classification problems. The same classification problem with WM data resulted in a low and insignificant balanced accuracy of 43.18% (*p* > .66; sensitivity: 69.70%, specificity: 16.67%).

**Table 3:** Weight averages by included region

Rank	Case‐classification					Disorder‐classification				
	Area	Side	Weight abs.	Weight perc.	Voxels	Area	Side	Weight abs.	Weight perc.	Voxels
1	Putamen	R	0.008225	7.36	693	Superior frontal gyrus	L	0.005132	5.33	2,426
2	Amygdala	R	0.006528	5.84	248	Middle frontal gyrus	R	0.005091	5.48	3,906
3	Middle frontal gyrus, orbital	R	0.005307	4.75	915	Inferior frontal gyrus, pars triangularis	R	0.005011	5.40	1,593
4	Middle frontal gyrus	R	0.004982	4.46	3,811	Inferior frontal gyrus, pars opercularis	R	0.004954	5.33	1,077
5	Putamen	L	0.004955	4.44	652	Nucleus caudate	R	0.004559	4.91	934
6	Nucleus caudate	R	0.004718	4.22	922	Inferior frontal gyrus, pars triangularis	L	0.004533	4.88	1,858
7	Superior frontal gyrus, orbital	R	0.004607	4.12	943	Superior frontal gyrus	R	0.004340	4.67	1,691
8	Inferior frontal gyrus, pars opercularis	R	0.004562	4.08	1,074	Middle frontal gyrus	L	0.004138	4.46	3,344
9	Amygdala	L	0.004442	3.98	220	Superior medial frontal gyrus	L	0.004030	4.34	2,287
10	Middle frontal gyrus	L	0.004413	3.95	3,307	Superior frontal gyrus	L	0.003939	4.24	2,898
11	Superior frontal gyrus	L	0.003974	3.56	2,389	Inferior frontal gyrus, orbital	R	0.003661	3.94	1,523
12	Superior frontal gyrus	R	0.003886	3.48	2,806	Middle frontal gyrus, orbital	L	0.003616	3.89	762
13	Anterior cingulate gyrus	L	0.003604	3.23	1,396	Amygdala	R	0.003056	3.29	248
14	Nucleus caudate	L	0.003440	3.08	857	Inferior frontal gyrus, orbital	L	0.003012	3.24	1,529
15	Inferior frontal gyrus, pars triangularis	R	0.003423	3.06	1,563	Inferior frontal gyrus, pars opercularis	L	0.002931	3.16	806
16	Hippocampus	L	0.003387	3.03	918	Anterior cingulate gyrus	R	0.002812	3.03	1,307
17	Inferior frontal gyrus, pars opercularis	L	0.003373	3.02	800	Medial frontal gyrus, orbital	R	0.002793	3.01	929
18	Hippocampus	R	0.003352	3.00	944	Superior frontal gyrus, orbital	L	0.002753	2.96	934
19	Inferior frontal gyrus, orbital	R	0.003259	2.92	1,511	Amygdala	L	0.002355	2.54	220
20	Inferior frontal gyrus, pars triangularis	L	0.002973	2.66	1829	Putamen	R	0.002345	2.52	811
21	Superior medial frontal gyrus	R	0.002962	2.65	1,676	Nucleus caudate	L	0.002193	2.36	868
22	Middle frontal gyrus, orbital	L	0.002909	2.60	758	Hippocampus	L	0.002095	2.26	918
23	Anterior cingulate gyrus	R	0.002835	2.54	1,304	Anterior cingulate gyrus	L	0.001897	2.04	1,396
24	Thalamus	L	0.002756	2.47	628	Superior frontal gyrus, orbital	R	0.001869	2.01	954
25	Superior medial frontal gyrus	L	0.002705	2.42	2,273	Medial frontal gyrus, orbital	L	0.001861	2.00	662
26	Inferior frontal gyrus, orbital	L	0.002517	2.25	1,520	Medial frontal gyrus, orbital	R	0.001810	1.95	824
27	Superior frontal gyrus, orbital	L	0.002093	1.87	931	Putamen	L	0.001670	1.80	706
28	Medial frontal gyrus, orbital	R	0.002088	1.87	823	Hippocampus	R	0.001664	1.79	945
29	Thalamus	R	0.001953	1.75	724	Thalamus	L	0.001555	1.67	628
30	Medial frontal gyrus, orbital	L	0.001491	1.33	653	Thalamus	R	0.001193	1.28	765

L, left side; R, right side. Area, brain region according to the automated anatomical labeling atlas (Tzourio‐Mazoyer et al., 2002); Weight abs., mean of absolute weight values of included all voxels within the region (Schrouff, Cremers, et al., 2013); Weight perc., percentage of normalized weight values of this region relative to the sum of all weight values of all regions for the respective classification problem. Voxels, number of voxels included.

Combining data from all four modalities using WAVE resulted in a balanced accuracy of 90.10% (*p* < .001) with 84.38% sensitivity and 95.83% specificity. Classifiers based on clinical questionnaire data were weighted significantly higher than classifiers based on neuroimaging and hormonal data (mean‐weights: clinical data: 0.56, GM data: 0.21, WM data: 0.13, cortisol data: 0.10; all *p*s < .001).

### 3.3 Disorder‐classification

Classification of GAD versus MD subjects using clinical questionnaire information resulted in a poor solution (balanced accuracy: 56.58%, *p* > .22, sensitivity: 63.16%, specificity: 50.00%). Here, particularly IUS‐12 scores provided information for classification (Table 2). In contrast, applying the cortisol data to the same classification problem resulted in good balanced accuracy (74.60%, *p* = .0088, sensitivity: 77.78%, specificity: 71.43%).

Disorder‐classification using GM data resulted in 68.05% balanced accuracy (*p* = .034, sensitivity: 78.95%, specificity: 57.14%). For this classification problem, the putamen and amygdala contributed less information while various frontal areas were of greater importance. Classification using WM data was less accurate (balanced accuracy: 58.27%, *p* > .15; sensitivity: 73.68%, specificity: 42.86%).

Combining data from all four modalities using WAVE resulted in a nominally lower balanced accuracy of 67.46% (*p* = .0268, sensitivity: 77.78%, specificity: 57.14%) than cortisol or GM accuracy alone. Classifiers based on cortisol data and GM data were weighted significantly higher than classifiers based on WM and clinical questionnaire data (*p*s < .001), which were of comparable size (*p* = .579). Classifiers based on cortisol data were also weighted significantly higher than classifiers based on GM data (*p* < .001; mean‐weights: clinical data: 0.17, cortisol data: 0.37, GM data: 0.28, WM data: 0.18).

Results from the additional analyses using whole‐brain data were less accurate in classification than the results based on ROIs for separate classifiers but comparable for the combined approach. Please see Appendix S2 for further details.

## 4 Discussion

Generalized anxiety disorder is a common and impairing disorder but recognition, diagnosis and differentiation from depression is a well‐known problem hampering treatment decisions. This proof‐of‐concept paper explored whether classifying subjects according to their clinical, hormonal, or neurostructural correlates yields accuracy rates beyond chance level, and whether combining data from different modalities improves classification. In a first analysis, subjects with a disorder and healthy comparison subjects were used to classify cases, whereas in a second analysis, subject with GAD and/or MD were used to classify GAD. The following main results emerged: (1) For case‐classification, clinical questionnaire scores clearly outperformed cortisol and MRI data, which yielded only poor results. (2) For the disorder‐classification, however, an inverse pattern emerged, with clinical questionnaire scores performing only on chance level and being outperformed by cortisol and MRI data, thus implying distinct (neuro‐)biological correlates for GAD and MD.

### 4.1 Differentiating cases from non‐cases (case‐classification)

Classification based on clinical data yielded very good results for case‐classification but only results on chance level for disorder‐classification. This result is also what would be expected when inspecting the basic sample characteristics. Clinical questionnaire scores were significantly different between the clinical groups and the HC group. Classification weights suggest that the STAI‐T scores provided most information for case‐classification, and indeed STAI‐T scores show a combination of high mean differences between the clinical and HC groups and relatively small standard deviations, resulting in the most marked difference overall. However, as this predictive approach is multivariate in nature, caution should be taken when interpreting singular features, as only the entire pattern holds predictive accuracy.

Contrary to clinical data, case‐classification using cortisol and MRI data yielded only poor results. Nonsignificant results were achieved for GM data, WM data, and cortisol. This finding is in line with previous research. Previous studies reported differences between GAD or MDD compared to healthy controls for cortisol (Bhagwagar et al., 2005; Hek et al., 2013; Hinkelmann et al., 2012; Mantella et al., 2008; Phillips et al., 2011; Steudte et al., 2011; Ulrike, Reinhold, & Dirk, 2013; Vreeburg et al., 2009; Wei et al., 2015) but the exact nature of these differences was mixed and some studies did not find such differences (Burke, Davis, Otte, & Mohr, 2005; see also the meta‐analysis by Vythilingam et al., 2004). The heterogeneity of prior studies can be attributed to methodological differences in data collection (e.g. diurnal profiles, awakening response, and stress response) or sample characteristics (e.g. age or comorbidities). We here assessed the cortisol release in a 100 min window during the experimental investigation. Because MRI scanning can be perceived as stressful situation, including a stress‐related cortisol reaction (Muehlhan, Lueken, Wittchen, & Kirschbaum, 2011), our data are rather comparable to a challenging situation instead of “baseline” release or diurnal profiles. Few data are available for GAD patients exposed to challenging situations. A study in adolescents with different anxiety disorders including GAD indicates no differences between GAD and healthy subjects as well (Gerra et al., 2000). The result was contrary to hypotheses for MRI data as brain anatomical differences in GAD and MD in areas such as the amygdala or parts of the basal ganglia have been repeatedly reported (Bora, Fornito, et al., 2012; Bora, Harrison, et al. 2012; Hilbert et al., 2014; Kempton et al., 2011). These areas were also indicated as most important for case‐classification in this study. The inability of the SVM to classify cases versus noncases based on cortisol and structural imaging data with better accuracy than questionnaire data alone might be related to the fact that subjects with different mental disorders were accumulated in one group. SVMs are linear classification algorithms. The inclusion of subjects with different disorders in one group also leads to the inclusion of brain scans with anatomical changes in different directions, for example in the case of GAD and MD increased and decreased GM volumes in certain frontal areas. The difficulty of finding a linear decision function to reliably separate these both disorder groups with their partly diverging abnormalities from the mean of HC subjects might explain the poor results achieved for the classification using MRI data here and likewise apply to the cortisol data.

### 4.2 Differentiating GAD from MD (disorder‐classification)

Separation of GAD and MD subjects based on clinical questionnaire data only resulted in poor accuracy. GAD and MD groups were very comparable regarding the range of questionnaire scores with significant differences being present only in the IUS‐12. Hence, the IUS‐12 most prominently contributed to the disorder‐classification. This is in line with the hypotheses derived from studies reporting that GAD is difficult to diagnose in primary care settings (Calleo et al., 2009; Munk‐Jorgensen et al., 2006; Vermani et al., 2011; Wittchen et al., 2002). We are not aware of studies in more specialized settings or in settings using more standardized diagnostic instruments, where diagnostic classification may be more accurate. On the other hand, while both the PSWQ and IUS‐12 measure constructs closely related to GAD, neither worrying nor intolerance of uncertainty are exclusively related to GAD but also present in MD (Carleton et al., 2012; Chelminski & Zimmerman, 2003; Gentes & Ruscio, 2011; Starcevic, 1995). Screening questionnaires designed specifically for GAD such as the GAD‐7, the ASQ or dimensional ratings such as the dimensional anxiety scales for DSM‐5 (dimensional scale for GAD: GAD‐D; Beesdo‐Baum et al., 2012; Lebeau et al., 2012) may therefore be better suited for the task of detecting GAD and might have supported diagnostic classification. Generally, the integration of 14 studies in a meta‐analysis by Plummer, Manea, Trepel, and McMillan (2016) indicated good sensitivity and specificity for the detection of GAD in different settings. Clinical interviews such as the SCID (First, Spitzer, Gibbon, & Williams, 1997) or MINI (Sheehan et al., 1998) served as reference. Fewer data are available for the GAD‐D for which good sensitivity but only moderate specificity have been reported (Beesdo‐Baum et al., 2012). Particularly screening instruments such as the GAD‐7 might therefore have provided additional information beyond the PSWQ and IUS‐12.

Classifiers based on cortisol and MRI data performed better for the disorder‐classification. Correct GAD classification rates of 74.60%, 68.05%, and 58.27% were achieved for cortisol, GM, and WM data. While these accuracies are not sufficient for clinical use at this stage and the proof‐of‐concept nature of this study has to be kept in mind as well, these findings still provide first evidence that (neuro‐)biological markers may provide incremental value supplementing clinical information. Accuracy for disorder‐classification is overall comparable to the results of proof‐of‐concept studies based on structural MRI data in other mental disorders such as MD which were reported to range from 67.6% to 90% (Costafreda, Chu, Ashburner, & Fu, 2009; Mwangi, Ebmeier, Matthews, & Steele, 2012; Patel et al., 2015). It is important to note that classification in this study was successful although a substantial proportion of GAD patients exhibited a comorbid depressive disorder. Inspection of the features related to classification revealed for cortisol that GAD subjects showed significantly lower cortisol release during the investigation. This is in line with the meta‐analysis by Burke et al. (2005) indicating cortisol release in response to psychological stress in MD being comparable to healthy subjects but contrary to a report indicating also comparable cortisol release in GAD (Gerra et al., 2000). However, interpretation of the GAD result is somewhat difficult as this is the only study on cortisol release in response to stress in GAD but it consisted only of adolescent subjects and also included other anxiety disorders besides GAD. To our best knowledge, cortisol data has not been used to support classification individual subjects so far.

Inspection of the brain areas associated with classification accuracy based on GM data suggests that mainly frontal and prefrontal areas provided information for differentiating GAD and MD. There is no study directly comparing GM volumes in pure GAD and pure MD, but results from work investigating structural correlates within one of the disorders suggest decreased GM volume in frontal areas for MD (Bora, Fornito, et al., 2012; Bora, Harrison, et al. 2012; Du et al., 2012; Kempton et al., 2011; Sacher et al., 2012), whereas increased GM volume has been reported for GAD (Schienle, Ebner, & Schafer, 2011). Results for GAD are inconsistent (Hilbert et al., 2014).

Findings from separate classification were overall in line with the previous literature: clinical information performed well for case‐classification but not for disorder‐classification, while the reversed pattern was found for cortisol and MRI data. As a consequence, it seemed reasonable to combine both types of information. Accuracy rates resulting from this combined approach were comparable to accuracy rates resulting from the best respective modality. The combined approach classified more than ninety percent of all subjects correctly as cases and noncases and about two‐thirds of all clinical subjects correctly as GAD subjects or pure MD subjects. Additionally, for both the case‐classification and the disorder‐classification, sensitivity was higher than specificity, that is most cases and GAD subjects were recognized as such. This is advantageous given the consequences which would follow this decision under real‐world circumstances, such as intervention. These results indicate that diagnostic markers based on biological information such as cortisol levels or brain anatomy might be helpful for complementing clinical data in the future, mostly in situations where classification is difficult.

### 4.3 Limitations

There are limitations to the results obtained in this study. The aim of this proof‐of‐concept paper was to demonstrate how (neuro‐)biological data might provide incremental value for the classification of GAD on an individual subject level. The results warrant further attention but do also indicate the need for improving accuracy rates in future studies. At the moment, the potential biomarkers investigated here would likely be outperformed by standardized clinical interviews in terms of accuracy and cost‐efficiency. Still, biomarkers bear potential for their usage in clinical contexts of mental disorders as, for example first studies provided promising findings for the prediction of treatment outcomes (Hahn et al., 2015; Levine, Rabinowitz, Uher, & Kapur, 2015; Uher, Tansey, Malki, & Perlis, 2012). The sample size was small in this study, groups were unbalanced and GAD subjects had to be separated only from one other related disorder. Future studies should try to employ larger balanced samples and might want to include other disorders that share some characteristics with GAD as well, such as other anxiety disorders or somatoform disorders. This way, the task of recognizing GAD and separating GAD from other disorders would be harder and show more resemblance to the task in real‐world clinical settings. While the inclusion of comorbidity in general made the clinical groups more heterogeneous and might therefore have reduced the accuracy of the classification, it also enhanced the similarity of the study samples to clinical GAD populations and therefore ensures the ecological validity of this investigation. Testing the classifiers on a second and independent dataset instead of in a LOOCV scheme would additionally increase the degree to which results can be generalized and are externally valid. Classification accuracy might also be improved by including specific screening instruments for GAD such as the GAD‐7 or GAD‐D in the questionnaire modality or by including more sophisticated methods of measuring WM characteristics such as diffusion tensor imaging in neuroimaging modality (Wen, Steffens, Chen, & Zainal, 2014). Furthermore, future studies could include further modalities such as functional MRI data, (epi)genetic data, or behavioral data.

## 5 Conclusions

In this proof‐of‐concept study, we investigated the ability to accurately classify subjects according to the presence of a mental disorder and according to the presence of GAD using clinical questionnaire data, cortisol data, and structural MRI data. Results showed that cortisol and MRI data were particularly able to provide incremental value to the disorder‐classification of GAD subjects beyond clinical questionnaire data alone. Classification based on combined data resulted in significant accuracy rates as well. Thus it seems possible that MRI data might be able to facilitate the correct diagnosis of GAD in the future. Further research on this question is warranted.

## Conflict of Interest

None declared.
