# Supplementary material for: Separating generalized anxiety disorder from major depression using clinical, hormonal, and structural MRI data: A multimodal machine learning study
Source: Brain Behav. 2017 Feb 12;7(3):e00633. doi: 10.1002/brb3.633 (PMC5346520; doi:10.1002/brb3.633)
Supplement: Supplementary file 1 [file BRB3-7-e00633-s001.doc]

**Appendix S1: Comorbidity**

Comorbid disorders within the clinical groups: GAD (including GAD only and comorbid GAD) and MD. Subjects were diagnosed using the Composite International Diagnostic Interview (CIDI; Wittchen and Pfister, 1997) and confirmation by clinical experts.

| **Supplemental Table 1.** Comorbid disorders according to DSM-IV. | | | | | |
| --- | --- | --- | --- | --- | --- |
|  | GAD (*n* = 19) | | MD (*n* = 14) | | |
| *Comorbid depressive disorders* | | | | | |
| Major Depression (*n*, %) | 12 | (63.16) | -- | -- | |
| Dysthymia (*n*, %) | 2 | (10.53) | -- | -- | |
| *Comorbid anxiety disorders* | | | | | |
| Social anxiety disorder (*n*, %) | 5 | (26.32) | -- | -- | |
| Specific phobia (*n*, %) | 4 | (21.05) | 1 | (7.14) | |
| Panic disorder with agoraphobia (*n*, %) | 1 | (5.26) | -- | -- | |
| Panic disorder without agoraphobia (*n*, %) | 1 | (5.26) | -- | -- | |
| Agoraphobia without history of panic disorder (*n*, %) | 1 | (5.26) | -- | -- | |
| Anxiety disorder not otherwise specified (*n*, %) | 1 | (5.26) | -- | -- | |
| *Other comorbid disorders* | | | | | |
| Pain disorder (*n*, %) | 3 | (15.79) | -- | -- | |
| Anorexia nervosa (*n*, %) | 1 | (5.26) | -- | -- | |
| Atypical Bulimia nervosa & Bulimia nervosa (*n*, %) | -- | -- | 2 | (14.29) | |
| Other developmental disorders of speech and language (*n*, %) | -- | -- | 1 | (7.14) | |
| Hypochondria (*n*, %) | 1 | (5.26) | 1 | (7.14) | |
| GAD: Generalized Anxiety Disorder subjects, including GAD only and comorbid GAD subjects; MD: Major Depression subjects. | | | | |  |

**Appendix S2: Pattern recognition based on whole-brain data**

Pattern recognition based on whole-brain data for grey (gm) and white matter (wm) and integration with the clinical and cortisol data were additionally conducted as supplemental analysis. The same data as in the main analyses were used. As a consequence, preprocessing was identical. Also similar to the main analyses, pattern recognition analyses were conducted once for identifying cases versus noncases and once for recognizing GAD versus MD subjects. Pattern recognition was done using PRoNTo (http://www.mlnl.cs.ucl.ac.uk/pronto/; Schrouff et al., 2013b). For both gm and wm, a mask was used to restrict analyses to voxels for which data were present in every subject. No further mask was applied to restrict analyses to certain ROIs however. Similar to the main analyses SVMs were used on mean centered and normalized data in an LOOCV framework. Balanced accuracy, sensitivity and specificity were calculated. Statistical significance was assessed by permutation tests based on 5000 iterations. Again, weight-maps and rank-orders of the regional weight averages were calculated for the GM data as described in (Schrouff et al., 2013a). Regions were anatomically defined according to the automated anatomical labeling atlas (aal; Tzourio-Mazoyer et al., 2002) as implemented in the wfu pickatlas toolbox (Maldjian et al., 2004, Maldjian et al., 2003).

Integration of the different classifiers was done similar to the main analyses. Weight-adjusted voting for ensembles of classifiers (WAVE; Kim et al., 2011) was used and classifier-weights were calculated in a nested LOOCV scheme. Please see the main body of the manuscript for further details. For the purpose of integrating the different classifiers, the classifier based on clinical and cortisol data from the main analyses was used. Integration of classifiers was conducted after excluding the subject with missing cortisol data as in the main analyses.

| **Supplemental table 2.** Weight averages by region for gm (whole-brain). | | | | | | | | | | |
| --- | --- | --- | --- | --- | --- | --- | --- | --- | --- | --- |
|  | Classification cases versus noncases | | | | | Classification GAD subjects versus MD subjects | | | | |
| Rank | Area | Side | weight abs. | weight perc. | voxels | Area | Side | weight abs. | weight perc. | voxels |
| 1 | putamen | R | 0.005862 | 3.23 | 693 | paracentral lobule | L | 0.005860 | 3.51 | 923 |
| 2 | pallidum | R | 0.003399 | 1.87 | 44 | paracentral lobule | R | 0.004254 | 2.55 | 666 |
| 3 | nucleus caudate | R | 0.003077 | 1.70 | 922 | Supplementary motor area | R | 0.003608 | 2.16 | 1950 |
| 4 | inferior frontal gyrus, pars opercularis | R | 0.003008 | 1.66 | 1074 | posterior cingulate gyrus | L | 0.003447 | 2.07 | 385 |
| 5 | putamen | L | 0.002990 | 1.65 | 652 | Supplementary motor area | L | 0.003138 | 1.88 | 1770 |
| 6 | paracentral lobule | R | 0.002963 | 1.63 | 656 | superior parietal gyrus | R | 0.002977 | 1.78 | 1243 |
| 7 | amygdala | R | 0.002907 | 1.60 | 248 | middle frontal gyrus | R | 0.002897 | 1.74 | 3906 |
| 8 | anterior cingulate gyrus | L | 0.002717 | 1.50 | 1396 | inferior frontal gyrus, pars triangularis | L | 0.002779 | 1.67 | 1858 |
| 9 | middle frontal gyrus | R | 0.002697 | 1.49 | 3811 | inferior frontal gyrus, pars triangularis | R | 0.002766 | 1.66 | 1593 |
| 10 | middle temporal pole | R | 0.002552 | 1.41 | 855 | superior parietal gyrus | L | 0.002755 | 1.65 | 1481 |
| 11 | superior parietal gyrus | R | 0.002544 | 1.40 | 1203 | precentral | R | 0.002727 | 1.63 | 2404 |
| 12 | middle temporal pole | L | 0.002472 | 1.36 | 581 | inferior parietal gyrus | L | 0.002673 | 1.60 | 2221 |
| 13 | cuneus | L | 0.002451 | 1.35 | 1409 | inferior frontal gyrus, pars opercularis | R | 0.002641 | 1.58 | 1077 |
| 14 | middle occipital gyrus | R | 0.002359 | 1.30 | 1803 | middle temporal pole | R | 0.002570 | 1.54 | 866 |
| 15 | calcarine sulcus | L | 0.002358 | 1.30 | 2121 | superior occipital gyrus | R | 0.002566 | 1.54 | 1204 |
| 16 | paracentral lobule | R | 0.002355 | 1.30 | 907 | middle frontal gyrus | L | 0.002510 | 1.50 | 3344 |
| 17 | postcentral gyrus | R | 0.002348 | 1.29 | 2659 | precentral | L | 0.002351 | 1.41 | 2667 |
| 18 | middle frontal gyrus | L | 0.002321 | 1.28 | 3307 | inferior temporal gyrus | L | 0.002334 | 1.40 | 3047 |
| 19 | nucleus caudate | L | 0.002321 | 1.28 | 857 | superior temporal pole | R | 0.002302 | 1.38 | 921 |
| 20 | calcarine sulcus | R | 0.002316 | 1.28 | 1508 | rolandic operculum | L | 0.002298 | 1.38 | 921 |
| 21 | precentral | L | 0.002314 | 1.28 | 2567 | nucleus caudate | R | 0.002229 | 1.34 | 934 |
| 22 | precentral | R | 0.002311 | 1.27 | 2275 | middle temporal gyrus | L | 0.002164 | 1.30 | 4590 |
| 23 | cerebellum | R | 0.002306 | 1.27 | 8106 | heschl’s gyrus | L | 0.002153 | 1.29 | 222 |
| 24 | insula | R | 0.002263 | 1.25 | 1710 | gyrus rectus | L | 0.002146 | 1.29 | 828 |
| 25 | superior temporal pole | R | 0.002254 | 1.24 | 902 | precuneus | L | 0.002111 | 1.27 | 2987 |
| 26 | lingual gyrus | L | 0.002234 | 1.23 | 2043 | middle cingulate gyrus | L | 0.002069 | 1.24 | 1926 |
| 27 | gyrus rectus | R | 0.002193 | 1.21 | 742 | middle temporal pole | L | 0.002012 | 1.22 | 605 |
| 28 | middle frontal gyrus, orbital | R | 0.002144 | 1.18 | 915 | superior frontal gyrus, orbital | L | 0.002030 | 1.22 | 934 |
| 29 | inferior r parietal gyrus | R | 0.002102 | 1.16 | 1095 | angular gyrus | R | 0.001994 | 1.19 | 1492 |
| 30 | superioparietal gyrus | L | 0.002098 | 1.16 | 1454 | supramarginal gyrus | L | 0.001972 | 1.18 | 1213 |
| 31 | superior occipital gyrus | L | 0.002088 | 1.15 | 1111 | middle occipital gyrus | L | 0.001965 | 1.18 | 3025 |
| 32 | fusiform gyrus | L | 0.002075 | 1.14 | 2274 | inferior parietal gyrus | R | 0.001959 | 1.17 | 1131 |
| 33 | parahippocampal gyrus | R | 0.002075 | 1.14 | 1124 | middle cingulate gyrus | R | 0.001949 | 1.17 | 2147 |
| 34 | rolandic operculum | L | 0.002069 | 1.14 | 912 | cerebellum | L | 0.001942 | 1.16 | 8289 |
| 35 | lingual gyrus | R | 0.002053 | 1.13 | 2116 | lingual gyrus | R | 0.001938 | 1.16 | 2060 |
| 36 | postcentral gyrus | L | 0.002044 | 1.13 | 2904 | supramarginal gyrus | R | 0.001931 | 1.16 | 1781 |
| 37 | inferior frontal gyrus, pars opercularis | L | 0.002040 | 1.12 | 800 | middle frontal gyrus, orbital | R | 0.001900 | 1.14 | 929 |
| 38 | middle temporal gyrus | L | 0.002039 | 1.12 | 4571 | middle occipital gyrus | R | 0.001886 | 1.13 | 1832 |
| 39 | cerebellum | L | 0.002027 | 1.12 | 8159 | superior temporal gyrus | R | 0.001836 | 1.10 | 2728 |
| 40 | superior temporal gyrus | R | 0.001971 | 1.09 | 2700 | superior frontal gyrus | R | 0.001780 | 1.07 | 2898 |
| 41 | superior frontal gyrus | L | 0.001958 | 1.08 | 2389 | middle frontal gyrus, orbital | L | 0.001770 | 1.06 | 762 |
| 42 | middle temporal gyrus | R | 0.001948 | 1.07 | 3876 | middle temporal gyrus | R | 0.001764 | 1.06 | 3989 |
| 43 | Supplementary motor area | R | 0.001931 | 1.06 | 1944 | superior frontal gyrus | L | 0.001758 | 1.05 | 2426 |
| 44 | angular gyrus | L | 0.001930 | 1.06 | 1121 | fusiform gyrus | L | 0.001757 | 1.05 | 2276 |
| 45 | supramarginal gyrus | R | 0.001917 | 1.06 | 1757 | anterior cingulate gyrus | R | 0.001749 | 1.05 | 1307 |
| 46 | inferior frontal gyrus, pars triangularis | R | 0.001906 | 1.05 | 1563 | superior temporal gyrus | L | 0.001725 | 1.03 | 2194 |
| 47 | inferior temporal gyrus | L | 0.001894 | 1.04 | 3004 | inferior frontal gyrus, pars opercularis | L | 0.001721 | 1.03 | 806 |
| 48 | angular gyrus | R | 0.001891 | 1.04 | 1455 | posterior cingulate gyrus | R | 0.001714 | 1.03 | 237 |
| 49 | superior frontal gyrus | R | 0.001890 | 1.04 | 2806 | inferior temporal gyrus fusiform gyrus | R | 0.001655 | 0.99 | 2466 |
| 50 | heschl’s gyrus | R | 0.001888 | 1.04 | 243 | postcentral gyrus | L | 0.001647 | 0.99 | 2976 |
| 51 | insula | L | 0.001879 | 1.04 | 1731 | gyrus rectus | R | 0.001635 | 0.98 | 742 |
| 52 | inferior temporal gyrus | R | 0.001879 | 1.04 | 3141 | angular gyrus | L | 0.001624 | 0.97 | 1132 |
| 53 | superior frontal gyrus, orbital | R | 0.001878 | 1.04 | 943 | cerebellum | R | 0.001615 | 0.97 | 8164 |
| 54 | fusiform gyrus | R | 0.001872 | 1.03 | 2464 | inferior occipital gyrus | L | 0.001607 | 0.96 | 915 |
| 55 | inferior parietal gyrus | L | 0.001822 | 1.00 | 2202 | rolandic operculum | R | 0.001573 | 0.94 | 1208 |
| 56 | precuneus | R | 0.001780 | 0.98 | 2779 | superior temporal pole | L | 0.001564 | 0.94 | 958 |
| 57 | pallidum | L | 0.001779 | 0.98 | 56 | inferior temporal gyrus | R | 0.001563 | 0.94 | 3192 |
| 58 | inferior frontal gyrus, pars triangularis | L | 0.001774 | 0.98 | 1829 | cuneus | R | 0.001544 | 0.93 | 1349 |
| 59 | superior occipital gyrus | R | 0.001761 | 0.97 | 1169 | olfactory cortex | L | 0.001541 | 0.92 | 277 |
| 60 | Supplementary motor area | L | 0.001729 | 0.95 | 1754 | superior occipital gyrus | L | 0.001472 | 0.88 | 1155 |
| 61 | rolandic operculum | R | 0.001715 | 0.95 | 1204 | postcentral gyrus | R | 0.001437 | 0.86 | 2736 |
| 62 | precuneus | L | 0.001704 | 0.94 | 2977 | cuneus | L | 0.001430 | 0.86 | 1421 |
| 63 | supramarginal gyrus | L | 0.001642 | 0.91 | 1200 | inferior frontal gyrus, orbital | L | 0.001399 | 0.84 | 1529 |
| 64 | thalamus | L | 0.001612 | 0.89 | 628 | precuneus | R | 0.001362 | 0.82 | 2797 |
| 65 | middle frontal gyrus, orbital | R | 0.001585 | 0.87 | 823 | thalamus | R | 0.001349 | 0.81 | 765 |
| 66 | superior temporal gyrus | L | 0.001584 | 0.87 | 2191 | inferior frontal gyrus, orbital | R | 0.001337 | 0.80 | 1523 |
| 67 | vermis | L/R | 0.001575 | 0.87 | 1846 | lingual gyrus | R | 0.001334 | 0.80 | 2139 |
| 68 | cuneus | R | 0.001569 | 0.86 | 1321 | parahippocampal gyrus | L | 0.001326 | 0.80 | 947 |
| 69 | middle occipital gyrus | L | 0.001546 | 0.85 | 2983 | nucleus caudate | L | 0.001286 | 0.77 | 868 |
| 70 | amygdala | L | 0.001544 | 0.85 | 220 | parahippocampal gyrus | R | 0.001259 | 0.75 | 1124 |
| 71 | middle cingulate gyrus | L | 0.001525 | 0.84 | 1926 | superior frontal gyrus, orbital | R | 0.001226 | 0.74 | 954 |
| 72 | superior temporal pole | L | 0.001509 | 0.83 | 938 | superior medial frontal gyrus | L | 0.001104 | 0.66 | 2287 |
| 73 | middle cingulate gyrus | R | 0.001482 | 0.82 | 2143 | inferior occipital gyrus | R | 0.001104 | 0.66 | 854 |
| 74 | superior medial frontal gyrus | R | 0.001479 | 0.82 | 1676 | middle frontal gyrus, orbital | L | 0.001098 | 0.66 | 662 |
| 75 | middle frontal gyrus, orbital | L | 0.001465 | 0.81 | 758 | vermis | L/R | 0.001089 | 0.65 | 1852 |
| 76 | inferior frontal gyrus, orbital | R | 0.001445 | 0.80 | 1511 | insula | L | 0.001077 | 0.65 | 1737 |
| 77 | inferior occipital gyrus | R | 0.001422 | 0.78 | 807 | calcarine sulcus | L | 0.001070 | 0.64 | 2136 |
| 78 | hippocampus | R | 0.001401 | 0.77 | 944 | amygdala | L | 0.001068 | 0.64 | 220 |
| 79 | olfactory cortex | R | 0.001399 | 0.77 | 289 | putamen | R | 0.001054 | 0.63 | 811 |
| 80 | superior medial frontal gyrus | L | 0.001389 | 0.77 | 2273 | middle frontal gyrus, orbital | R | 0.001011 | 0.61 | 824 |
| 81 | anterior cingulate gyrus | R | 0.001333 | 0.73 | 1304 | thalamus | L | 0.000982 | 0.59 | 628 |
| 82 | hippocampus | L | 0.001309 | 0.72 | 918 | anterior cingulate gyrus | L | 0.000933 | 0.56 | 1396 |
| 83 | thalamus | R | 0.001282 | 0.71 | 724 | calcarine sulcus calcarine sulcus | R | 0.000911 | 0.55 | 1549 |
| 84 | inferior occipital gyrus | L | 0.001261 | 0.69 | 897 | insula | R | 0.000889 | 0.53 | 1722 |
| 85 | olfactory cortex | L | 0.001251 | 0.69 | 277 | calcarine sulcus | R | 0.000887 | 0.53 | 1691 |
| 86 | gyrus rectus | L | 0.001201 | 0.66 | 826 | olfactory cortex | L | 0.000862 | 0.52 | 289 |
| 87 | posterior cingulate gyrus | L | 0.001149 | 0.63 | 236 | putamen | L | 0.000821 | 0.49 | 706 |
| 88 | middle frontal gyrus, orbital | L | 0.001120 | 0.62 | 653 | hippocampus | L | 0.000759 | 0.46 | 918 |
| 89 | heschl’s gyrus | R | 0.001117 | 0.62 | 222 | pallidum | R | 0.000743 | 0.45 | 62 |
| 90 | parahippocampal gyrus | L | 0.001099 | 0.61 | 947 | heschl’s gyrus | R | 0.000688 | 0.41 | 243 |
| 91 | inferior frontal gyrus, orbital | L | 0.001036 | 0.57 | 1520 | hippocampus | R | 0.000661 | 0.40 | 945 |
| 92 | superior frontal gyrus, orbital | L | 0.000873 | 0.48 | 931 | amygdala | R | 0.000557 | 0.33 | 248 |
| 93 | posterior cingulate gyrus | L | 0.000743 | 0.41 | 385 | pallidum | L | 0.000284 | 0.17 | 75 |
| L: left side; R: right side. Area: brain region according to the automated anatomical labeling atlas (Tzourio-Mazoyer et al., 2002); Weight abs.: mean of absolute weight values of included all voxels within the region (Schrouff et al., 2013a); Weight perc.: Percentage of normalized weight values of this region relative to the sum of all weight values of all regions for the respective classification problem. Voxels: number of voxels included. | | | | | | | | | | |

| **Supplemental table 3.** Weight averages by region for wm (whole-brain). | | | | | | | | | | |
| --- | --- | --- | --- | --- | --- | --- | --- | --- | --- | --- |
|  | Classification cases versus noncases | | | | | Classification GAD subjects versus MD subjects | | | | |
| Rank | Area | Side | weight abs. | weight perc. | voxels | Area | Side | weight abs. | weight perc. | voxels |
| 1 | paracentral lobule | R | 0.007513 | 3.26 | 431 | angular gyrus | L | 0.004866 | 2.20 | 525 |
| 2 | paracentral lobule | L | 0.005340 | 2.31 | 643 | gyrus rectus | L | 0.004471 | 2.02 | 541 |
| 3 | superior parietal gyrus | L | 0.004772 | 2.07 | 559 | superior medial frontal gyrus | L | 0.004344 | 1.96 | 703 |
| 4 | inferior parietal gyrus | R | 0.004754 | 2.06 | 219 | middle temporal pole | L | 0.004186 | 1.89 | 218 |
| 5 | superior parietal gyrus | R | 0.004182 | 1.81 | 311 | paracentral lobule | L | 0.004094 | 1.85 | 672 |
| 6 | supramarginal gyrus | R | 0.004096 | 1.78 | 890 | inferior temporal gyrus | R | 0.003839 | 1.74 | 1576 |
| 7 | inferior occipital gyrus | L | 0.003933 | 1.70 | 612 | middle temporal pole | R | 0.003839 | 1.74 | 418 |
| 8 | middle frontal gyrus, orbital | L | 0.003925 | 1.70 | 409 | middle frontal gyrus | L | 0.003837 | 1.74 | 1453 |
| 9 | middle frontal gyrus | L | 0.003817 | 1.65 | 1420 | inferior frontal gyrus, orbital | L | 0.003827 | 1.73 | 838 |
| 10 | fusiform gyrus | L | 0.003685 | 1.60 | 1104 | paracentral lobule | R | 0.003740 | 1.69 | 436 |
| 11 | inferior frontal gyrus, orbital | L | 0.003665 | 1.59 | 834 | inferior parietal gyrus | L | 0.003720 | 1.68 | 888 |
| 12 | middle frontal gyrus | R | 0.003619 | 1.57 | 1451 | precuneus | L | 0.003574 | 1.62 | 1433 |
| 13 | supplementary motor area | R | 0.003618 | 1.57 | 1189 | inferior frontal gyrus, pars triangularis | R | 0.003565 | 1.61 | 948 |
| 14 | inferior frontal gyrus, orbital | R | 0.003527 | 1.53 | 718 | superior occipital gyrus | R | 0.003530 | 1.60 | 805 |
| 15 | superior medial frontal gyrus | L | 0.003500 | 1.52 | 1328 | inferior parietal gyrus | R | 0.003487 | 1.58 | 237 |
| 16 | Supplementary motor area | L | 0.003429 | 1.49 | 827 | Supplementary motor area | R | 0.003432 | 1.55 | 1225 |
| 17 | inferior temporal gyrus | L | 0.003378 | 1.46 | 1711 | middle frontal gyrus, orbital | R | 0.003367 | 1.52 | 420 |
| 18 | precentral | L | 0.003370 | 1.46 | 1740 | middle frontal gyrus | R | 0.003302 | 1.49 | 1493 |
| 19 | middle temporal pole | R | 0.003264 | 1.41 | 399 | inferior frontal gyrus, pars triangularis | L | 0.003272 | 1.48 | 1244 |
| 20 | inferior frontal gyrus, pars opercularis | L | 0.003205 | 1.39 | 531 | superior parietal gyrus | L | 0.003241 | 1.47 | 590 |
| 21 | middle occipital gyrus | R | 0.003169 | 1.37 | 924 | precentral | R | 0.003209 | 1.45 | 1742 |
| 22 | putamen | L | 0.003162 | 1.37 | 1009 | superior temporal pole | L | 0.003199 | 1.45 | 181 |
| 23 | angular gyrus | R | 0.003152 | 1.37 | 536 | inferior occipital gyrus | L | 0.003178 | 1.44 | 635 |
| 24 | pallidum | L | 0.003120 | 1.35 | 293 | inferior frontal gyrus, pars opercularis | R | 0.003025 | 1.37 | 616 |
| 25 | lingual gyrus | L | 0.003076 | 1.33 | 1405 | inferior temporal gyrus | L | 0.002927 | 1.32 | 1813 |
| 26 | inferior occipital gyrus | R | 0.003065 | 1.33 | 482 | middle temporal gyrus | L | 0.002920 | 1.32 | 2531 |
| 27 | superior occipital gyrus | L | 0.002989 | 1.30 | 834 | middle temporal gyrus | R | 0.002915 | 1.32 | 1812 |
| 28 | inferior frontal gyrus, pars triangularis | L | 0.002922 | 1.27 | 1235 | superior parietal gyrus | R | 0.002896 | 1.31 | 359 |
| 29 | precentral | R | 0.002908 | 1.26 | 1703 | cuneus | R | 0.002881 | 1.30 | 994 |
| 30 | angular gyrus | L | 0.002901 | 1.26 | 519 | superior frontal gyrus, orbital | R | 0.002839 | 1.28 | 1550 |
| 31 | supramarginal gyrus | L | 0.002871 | 1.24 | 647 | Supplementary motor area | L | 0.002805 | 1.27 | 861 |
| 32 | middle temporal gyrus | R | 0.002748 | 1.19 | 1722 | inferior frontal gyrus, pars opercularis | L | 0.002762 | 1.25 | 531 |
| 33 | precuneus | L | 0.002740 | 1.19 | 1396 | precentral | L | 0.002743 | 1.24 | 1793 |
| 34 | superior frontal gyrus, orbital | R | 0.002685 | 1.16 | 846 | cerebellum | L | 0.002737 | 1.24 | 3693 |
| 35 | parahippocampal gyrus | R | 0.002675 | 1.16 | 667 | middle occipital gyrus | R | 0.002730 | 1.23 | 985 |
| 36 | superior temporal pole | L | 0.002604 | 1.13 | 156 | postcentral gyrus | R | 0.002689 | 1.22 | 1789 |
| 37 | cerebellum | R | 0.002574 | 1.12 | 2947 | middle frontal gyrus, orbital | L | 0.002638 | 1.19 | 432 |
| 38 | middle occipital gyrus | L | 0.002565 | 1.11 | 2189 | angular gyrus cerebellum | R | 0.002629 | 1.19 | 2989 |
| 39 | superior frontal gyrus | L | 0.002529 | 1.10 | 688 | middle occipital gyrus | L | 0.002596 | 1.17 | 2302 |
| 40 | cuneus | L | 0.002475 | 1.07 | 683 | precuneus | R | 0.002559 | 1.16 | 1597 |
| 41 | inferior frontal gyrus, pars opercularis | R | 0.002475 | 1.07 | 615 | posterior cingulate gyrus | L | 0.002532 | 1.14 | 354 |
| 42 | postcentral gyrus | R | 0.002444 | 1.06 | 1749 | superior temporal gyrus | R | 0.002497 | 1.13 | 1668 |
| 43 | middle temporal gyrus | L | 0.002438 | 1.06 | 2389 | supramarginal gyrus | L | 0.002488 | 1.12 | 659 |
| 44 | inferior temporal gyrus | R | 0.002427 | 1.05 | 1488 | lingual gyrus | L | 0.002435 | 1.10 | 1436 |
| 45 | gyrus rectus | L | 0.002424 | 1.05 | 533 | calcarine sulcus | R | 0.002404 | 1.09 | 1554 |
| 46 | parahippocampal gyrus | L | 0.002392 | 1.04 | 563 | gyrus | R | 0.002385 | 1.08 | 563 |
| 47 | superior medial frontal gyrus | R | 0.002386 | 1.03 | 1538 | calcarine sulcus | L | 0.002330 | 1.05 | 1293 |
| 48 | amygdala | L | 0.002359 | 1.02 | 84 | rolandic operculum | R | 0.002287 | 1.03 | 766 |
| 49 | precuneus | R | 0.002288 | 0.99 | 1546 | fusiform gyrus | R | 0.002263 | 1.02 | 1192 |
| 50 | inferior frontal gyrus, pars triangularis | R | 0.002265 | 0.98 | 937 | olfactory cortex | L | 0.002251 | 1.02 | 175 |
| 51 | cerebellum | L | 0.002248 | 0.97 | 3609 | fusiform gyrus | L | 0.002223 | 1.01 | 1161 |
| 52 | fusiform gyrus | R | 0.002216 | 0.96 | 1139 | postcentral gyrus | L | 0.002116 | 0.96 | 2131 |
| 53 | rolandic operculum | R | 0.002202 | 0.95 | 726 | inferior frontal gyrus, orbital | R | 0.002101 | 0.95 | 737 |
| 54 | superior temporal gyrus | L | 0.002187 | 0.95 | 1127 | superior temporal gyrus | L | 0.002080 | 0.94 | 1152 |
| 55 | middle temporal pole | L | 0.002185 | 0.95 | 212 | superior medial frontal gyrus | R | 0.002033 | 0.92 | 647 |
| 56 | inferior parietal gyrus | L | 0.002184 | 0.95 | 838 | posterior cingulate gyrus | R | 0.001993 | 0.90 | 304 |
| 57 | lingual gyrus | L | 0.002137 | 0.93 | 1365 | parahippocampal gyrus | L | 0.001963 | 0.89 | 576 |
| 58 | gyrus rectus | R | 0.002103 | 0.91 | 534 | vermis | L/R | 0.001956 | 0.88 | 861 |
| 59 | calcarine sulcus | L | 0.002092 | 0.91 | 1235 | cuneus | L | 0.001879 | 0.85 | 699 |
| 60 | vermis | L/R | 0.002034 | 0.88 | 842 | supramarginal gyrus | R | 0.001846 | 0.83 | 944 |
| 61 | thalamus | L | 0.002024 | 0.88 | 969 | superior frontal gyrus, orbital | L | 0.001820 | 0.82 | 1358 |
| 62 | olfactory cortex | L | 0.001988 | 0.86 | 173 | middle frontal gyrus, orbital | L | 0.001805 | 0.82 | 346 |
| 63 | superior frontal gyrus | R | 0.001962 | 0.85 | 641 | insula | L | 0.001801 | 0.81 | 1055 |
| 64 | middle frontal gyrus, orbital | R | 0.001840 | 0.80 | 425 | superior temporal pole | R | 0.001798 | 0.81 | 234 |
| 65 | superior frontal gyrus, orbital | L | 0.001825 | 0.79 | 1131 | inferior occipital gyrus | R | 0.001773 | 0.80 | 504 |
| 66 | postcentral gyrus | L | 0.001816 | 0.79 | 2057 | anterior cingulate gyrus | R | 0.001764 | 0.80 | 826 |
| 67 | putamen | R | 0.001813 | 0.79 | 1064 | superior frontal gyrus | R | 0.001693 | 0.77 | 851 |
| 68 | heschl’s gyrus | R | 0.001709 | 0.74 | 85 | middle frontal gyrus, orbital | R | 0.001676 | 0.76 | 427 |
| 69 | insula | L | 0.001662 | 0.72 | 1018 | middle cingulate gyrus | R | 0.001641 | 0.74 | 1307 |
| 70 | cuneus | R | 0.001640 | 0.71 | 965 | superior frontal gyrus | L | 0.001639 | 0.74 | 1150 |
| 71 | superior occipital gyrus | R | 0.001639 | 0.71 | 767 | gyrus rectus | R | 0.001630 | 0.74 | 538 |
| 72 | middle frontal gyrus, orbital | R | 0.001592 | 0.69 | 412 | vermis putamen | R | 0.001623 | 0.73 | 1064 |
| 73 | heschl’s gyrus | R | 0.001560 | 0.68 | 96 | olfactory cortex | R | 0.001614 | 0.73 | 172 |
| 74 | superior temporal gyrus | R | 0.001560 | 0.68 | 1637 | superior occipital gyrus | L | 0.001602 | 0.72 | 854 |
| 75 | middle cingulate gyrus | R | 0.001530 | 0.66 | 1276 | putamen | L | 0.001550 | 0.70 | 1009 |
| 76 | calcarine sulcus | R | 0.001487 | 0.64 | 1462 | lingual gyrus | R | 0.001484 | 0.67 | 1417 |
| 77 | thalamus | R | 0.001476 | 0.64 | 947 | anterior cingulate gyrus | L | 0.001464 | 0.66 | 720 |
| 78 | amygdala | R | 0.001421 | 0.62 | 146 | middle cingulate gyrus | L | 0.001420 | 0.64 | 1019 |
| 79 | anterior cingulate gyrus | L | 0.001418 | 0.61 | 710 | thalamus | R | 0.001395 | 0.63 | 949 |
| 80 | superior temporal pole | R | 0.001414 | 0.61 | 232 | parahippocampal gyrus | R | 0.001316 | 0.60 | 672 |
| 81 | insula | R | 0.001376 | 0.60 | 768 | amygdala | L | 0.001301 | 0.59 | 94 |
| 82 | rolandic operculum | R | 0.001367 | 0.59 | 640 | insula | R | 0.001246 | 0.56 | 785 |
| 83 | olfactory cortex | R | 0.001357 | 0.59 | 169 | rolandic operculum | L | 0.001120 | 0.51 | 648 |
| 84 | posterior cingulate gyrus | R | 0.001326 | 0.57 | 303 | amygdala | R | 0.001097 | 0.50 | 158 |
| 85 | middle cingulate gyrus | L | 0.001272 | 0.55 | 1000 | nucleus caudate | R | 0.001039 | 0.47 | 738 |
| 86 | hippocampus | R | 0.001213 | 0.53 | 762 | pallidum | L | 0.001006 | 0.45 | 293 |
| 87 | middle frontal gyrus, orbital | L | 0.001187 | 0.51 | 341 | Pallidum | R | 0.000971 | 0.44 | 280 |
| 88 | anterior cingulate gyrus | R | 0.001139 | 0.49 | 819 | thalamus | L | 0.000921 | 0.42 | 969 |
| 89 | pallidum | R | 0.001054 | 0.46 | 280 | hippocampus | L | 0.000906 | 0.41 | 718 |
| 90 | hippocampus | L | 0.000927 | 0.40 | 715 | hippocampus | R | 0.000687 | 0.31 | 773 |
| 91 | posterior cingulate gyrus | L | 0.000833 | 0.36 | 346 | heschl’s gyrus | R | 0.000678 | 0.31 | 101 |
| 92 | nucleus caudate | L | 0.000718 | 0.31 | 632 | nucleus caudate | L | 0.000653 | 0.30 | 635 |
| 93 | nucleus caudate | R | 0.000534 | 0.23 | 732 | heschl’s gyrus | L | 0.000559 | 0.25 | 88 |
| L: left side; R: right side. Area: brain region according to the automated anatomical labeling atlas (Tzourio-Mazoyer et al., 2002); Weight abs.: mean of absolute weight values of included all voxels within the region (Schrouff et al., 2013a); Weight perc.: Percentage of normalized weight values of this region relative to the sum of all weight values of all regions for the respective classification problem. Voxels: number of voxels included. | | | | | | | | | | |

**Classification: Cases versus noncases**

Supplemental tables 2 and 3 show the averaged weights of the brain regions according to aal. Classifying cases versus noncases using whole-brain GM data resulted in a balanced accuracy of only 48.11% (sensitivity: 87.88%, specificity: 8.33%, p > 0.39). Similar to the main analyses, particularly the putamen and amygdala were important for the classification of cases versus noncases. The same classification problem with WM data resulted in a low and insignificant balanced accuracy of 45.27% (p > 0.56; sensitivity: 69.70%, specificity: 20.83%). Here, particularly wm volume in the paracentral lobule and the parietal and medial frontal areas provided information for classification.

Combining data from all three modalities using WAVE resulted in a balanced accuracy of 93.23% (p < 0.001), with 90.63% sensitivity and 95.83% specificity, comparable to the result from the main analysis. Classifier-weights suggested that this findings was related to the important role of the classifier based on clinical information, which did not change in this supplemental analysis (mean-weights: clinical data: 0.61, cortisol data: 0.11,gm data: 0.16, wm data: 0.12; all ps < 0.001 except cortisol vs wm data: p = 0.38).

**Classification: GAD versus MD**

The classification of GAD versus MD subjects using GM data resulted in 59.02 % balanced accuracy (sensitivity: 89.47%, specificity: 28.57%, p = 0.11). For this classification problem, the putamen and amygdala contributed less information while various frontal areas were of greater importance, comparable to the main analyses. Furthermore, gm volume in the paracentral lobule was ranked as providing important information for classification. Classification using WM data was not accurate (37.78%, p > 0.75; sensitivity: 68.42%, specificity: 7.14%). Again the paracentral lobule and additional temporal and frontal areas were important for the classification of GAD versus MD subjects.

Combining data from all three modalities using WAVE resulted in a balanced accuracy of 66.67% (p < 0.001) with 83.33% sensitivity and 50.00% specificity. Classifiers based on gm and clinical data were weighted significantly higher than the wm classifier (ps < 0.001; mean-weights: clinical data: 0.21, cortisol data: 0.44, gm data: 0.21, wm data: 0.14).

Overall, the findings of these supplemental analyses using whole-brain data for gm and wm demonstrate markedly less accurate classification results. These seemed particularly related to decreased specificity. On the other hand, inspection of the weight-maps and associated rankings showed that the most relevant brain areas were comparable to the main analyses for the majority of results. This indicates that most information added by the whole-brain approach was not valuable for the classification purpose.

When combining all classifiers the final decision still showed comparable accuracy to the results from the main analyses. Inspection of the classifier weights suggest that this finding was related to the marginalization of the influence of the gm and wm classifiers in favor of the clinical and cortisol classifiers, respectively.
